# Childhood Maltreatment and Revictimization: A Systematic Literature Review

**DOI:** 10.1177/15248380221150475

**Published:** 2023-02-03

**Authors:** Fatemeh Fereidooni, Judith K. Daniels, Miriam J. J. Lommen

**Affiliations:** 1 Department of Clinical Psychology and Experimental Psychopathology, University of Groningen, The Netherlands

**Keywords:** childhood maltreatment, childhood trauma, adulthood victimization, revictimization, repeated victimization

## Abstract

There is established evidence that childhood/adolescent victimization is associated with victimization in adulthood although the underlying mechanisms are not still clear. The current study aimed to systematically review empirical studies examining potential psychological factors linking childhood maltreatment to victimization in adulthood and the gaps in the literature. Following PRISMA protocol, 71 original studies consisting of a total sample of *n* = 31,633 subjects were analyzed. Symptom severity for various trauma-related disorders, dissociation, emotion dysregulation, and risky sexual behaviors emerged as potential predictors of revictimization. While these potential risk factors mediate the relationship between childhood maltreatment and adulthood victimization, evidence for additional factors such as social support, attachment styles, maladaptive schemas, and risk detection is very limited. Addressing these intrapersonal risk factors, found by prior studies, in interventions and preventive programs might decrease the probability of revictimization. The interactions between the identified risk factors have not been studied well yet. Hence, more research on mediating risk factors of revictimization is needed.

Childhood maltreatment (CM), defined as emotional, physical, sexual abuse, and emotional and physical neglect, has a high prevalence across the world. Approximately, three in four children experience physical and/or emotional victimization. For sexual abuse, this rate is one in five in women and 1 in 13 in men (World Health Organization, 2020). Previous studies provided extensive evidence in support of higher vulnerability for adulthood victimization (i.e., physical, emotional, and sexual abuse) among survivors of CM. Victimization both in childhood and adulthood is called *revictimization* in the literature and has received increasing attention in the last decades. A recent meta-analysis indicated that approximately half of individuals with a child sexual abuse history (CSA) are at risk of revictimization (Walker et al., 2019). Several potential mediators have been queried regarding their predictive value, such as dissociation (Zamir et al., 2018), posttraumatic stress disorder (PTSD) symptoms (Ullman & Peter-Hagene, 2016), and emotion dysregulation (Messman-Moore et al., 2013). Considering the increasing evidence for a strong link between CM and adulthood victimization, it is imperative to elucidate the psychological risk factors mediating revictimization. Therefore, it is important to review the findings of prior research to shed light on the factors examined so far, and the gaps in the literature.

Except for a recent non-systematic review (Walker & Wamser-Nanney, 2022), previous literature reviews were non-systematic, exclusively on sexual revictimization, and are now outdated (Arata, 2002; Breitenbecher, 2001; C. C. Classen, 2005; Lalor & McElvaney, 2010; Messman-Moore & Long, 2003). Hence, the aim of this study is to present the current empirical evidence regarding psychological associates of revictimization via a systematic review of the literature. The rationale for focusing on intrapersonal variables related to revictimization is to identify risk factors with predictive value that can be the focus of intervention or preventive programs as well as provide directions for future research. Since most studies so far have focused on a particular type of victimization or a specific population, this study aims to provide a general overview of the psychological risk factors by including all types of victimization and populations.

## Method

### Literature Search

The search was conducted following the PRISMA protocol (Moher et al., 2009). Two sets of search terms were selected, one for CM (“child abuse,” “child trauma,” “child maltreatment,” “incest,” “adverse childhood experiences,” “child neglect,” and “family violence”) and the other for revictimization (“revictimization,” “repeat victimization,” “polyvictimization,” “repeated trauma,” “multiple victimization,” “retraumatization,” “intimate partner violence,” “victimization,” “sexual aggression,” “sexual violence,” “rape,” “assault,” “domestic violence,” “betrayal trauma,” “adult victims,” and “dating violence”). The fully crossed combination of the two sets were searched in Psychinfo, PubMed, ScienceDirect, Springer, and Google Scholar from 2018 to the beginning of 2019. We stopped the search on Google Scholar, once there were no relevant hits on three consecutive pages.

Beforehand, we already had compiled several relevant studies and compared this compilation post hoc to the search results. We noticed that 11 topical studies were not detected by the systematic search due to their outlets not being represented in the searched databases. We, therefore, decided to additionally search the eight journals, which had published these studies, employing the same search terms. Finally, the references of included articles were screened for additional studies.

### Selection of Literature

**Figure 1.**
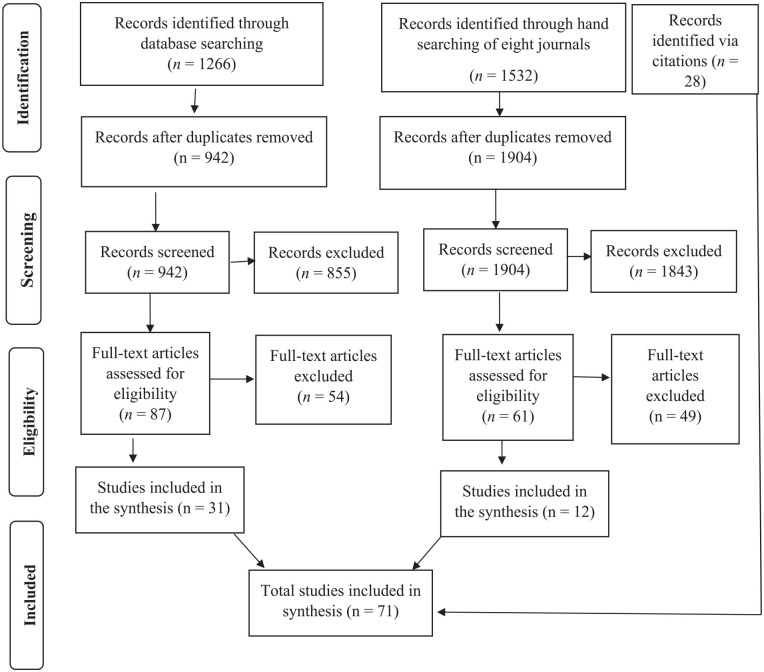
PRISMA 2009 flow diagram.

The inclusion criteria for the studies were (1) published in peer-reviewed journals, (2) quantitative research, (3) examining psychological associates of revictimization, and (4) clear definition of maltreatment in childhood/adolescence and adulthood victimization based on age ranges. The latter criterion became necessary as prior studies considered various developmental stages for revictimization. While most studies define revictimization as victimization in both childhood and adulthood (Arata, 2000; Babcock, 2012; Jankowski et al., 2002), more than one victimization experience in a lifetime, regardless of the age at occurrence, was defined as revictimization by other authors (Matlow & DePrince, 2013; Reichert et al., 2015). Due to the lack of research on the influence of these different age cut-offs on the relationship between revictimization and its risk factors, we cannot assume the results of these studies are comparable. Therefore, we limited our review to the studies that defined revictimization as interpersonal violence in both childhood/adolescence and adulthood. The flow diagram (see Figure 1) presents the procedure used for selecting eligible studies. One of the authors (F. F) and a research assistant screened the abstracts separately. Consensus between the two assessors about inclusion/exclusion of the studies was achieved for each abstract. Then, the author (F. F) examined each paper based on the inclusion criteria by assessing relevant information in the method and results sections of each paper. For quality assessment in this stage, the author (F.F) investigated the consistency between operational definition of revictimization and actual computation of revictimization. Papers not meeting the criteria were excluded from further analysis.

Afterward, information consisting of sample characteristics (e.g., sample size, population, gender, the country where the data were collected), design of the study, definition of CM/adulthood victimization, measures for tested predictors/correlates of revictimization, and results for each predictor/correlate were extracted from the included papers by the author. For the quality assessment in this stage, the author examined if *p*-value of at least .05 is used for each hypothesis, when *p*-value correction was not needed. Since we found studies on a wide variety of psychological risk factors, we opted to exclude the risk factors examined by only one study, which do not allow any conclusion or comparison with other studies. The excluded factors were social adjustment (Kaltman et al., 2005), shame (Kessler & Bieschke, 1999), psychological mindedness (Zamir & Lavee, 2014), partner selection (Gobin, 2012), psychophysical reaction to trauma-related stimuli (Patriquin et al., 2012), attentional bias to trauma-related stimuli (Field et al., 2001), and emotion recognition (Bell & Naugle, 2008). The final sample consisted of 71 studies. The results section is organized in a way that variables with more evidence are discussed at the top and within each paragraph, longitudinal studies are presented first due to their higher validity. Supplemental Table S1 in the Supplemental section provides information about the included papers.

## Results

### Mental Health

#### PTSD symptoms

Twenty-five papers examined PTSD (*n* = 6 longitudinal). Longitudinal studies provided preliminary evidence in favor of the role of PTSD symptoms in revictimization. A longitudinal study (Noll et al., 2003) found a significant relationship between lifetime PTSD severity and sexual/physical revictimization. Two of three prospective studies supported a mediating (Messman-Moore et al., 2005) or moderating (Sandberg et al., 1999) role of PTSD symptoms severity between childhood/adolescent sexual victimization and adulthood sexual victimization, while the study by Livingston et al. (2007) did not. In terms of specific PTSD symptoms, numbing was a mediator between CSA and prospective adulthood sexual victimization. Three symptom clusters of PTSD, re-experience, avoidance, and arousal, were intervening factors between CSA and prospective adult sexual victimization via problematic drinking (Ullman et al., 2009). Messman-Moore et al. (2009) provided evidence that PTSD severity was an intervening variable between CM and prospective rape in adulthood through risky sexual behavior/sexual dissatisfaction and substance/alcohol abuse.

Sexually revictimized individuals showed higher PTSD symptom levels than people who were sexually abused exclusively either in childhood or adulthood (Aosved et al., 2011; Arata, 1999a; Arata, 2000; Banyard et al., 2001; Bell & Naugle, 2008; Cloitre et al., 1997; Filipas & Ullman, 2006; Gibson & Leitenberg, 2001; Heidt et al., 2005; Kaltman et al., 2005; Messman-Moore et al., 2000; Schumm et al., 2006) and non-victims (Messman-Moore et al., 2000) in cross-sectional studies. Two studies found that revictimized women were more likely to meet diagnostic criteria for current PTSD than non-victims (Schumm et al., 2006) or for lifetime PTSD than sexually abused exclusively in childhood or adulthood and non-victims (Arata, 1999c), but not for current PTSD diagnosis (Arata, 1999c). No difference was found between revictimized women and women sexually victimized exclusively in adulthood (Bolstad & Zinbarg, 1997).

The cross-sectional studies indicated conflicting results. Mokma et al. (2016) found a significant indirect relationship between PTSD severity and sexual revictimization that is inconsistent with Filipas and Ullman (2006) and Bell and Naugle (2008) results. Dietrich (2007) reported a relationship between sexual revictimization and PTSD diagnosis only in women, but not in men. Walsh et al. (2013) and Risser et al. (2006) found that hyperarousal mediated the relationship between sexual victimization in childhood and adulthood.

#### Depression

Thirteen studies (*n* = 2 longitudinal) examined depression. A longitudinal study (Culatta et al., 2017) indicated that depression at the end of the first year of university mediated the relationship between sexual victimization in adolescence (i.e., at 14-years old) and over the second year of university. Livingston et al. (2007) found that depression was an intervening variable in the association between CSA and prospective adult sexual victimization.

Seven cross-sectional studies support the notion that revictimized subjects exhibit higher levels of depression than non-victims and, abused exclusively either in childhood or adulthood (Aosved et al., 2011, Banyard et al., 2001; Cloitre et al., 1997; Gidycz et al., 1993; Heidt et al., 2005; Kaltman et al., 2005; Messman-Moore et al., 2000; Schumm et al., 2006). However, this difference was not found for depression severity (Field et al., 2001) or for lifetime diagnosis of depressive disorders (Arata, 1999b) and depression severity was not related to sexual revictimization (C. Classen et al., 2002).

#### Anxiety

Eight cross-sectional studies investigated the role of anxiety. The studies found higher anxiety symptoms in sexually revictimized women than victimized exclusively in childhood or adulthood (Gidycz et al., 1993), and non-victims (Messman-Moore et al., 2000; Proulx et al., 1995). Three studies did not find higher lifetime prevalence of anxiety disorders (Arata, 1999b) or anxiety levels (Aosved et al., 2011; Field et al., 2001) in sexually revictimized individuals than non-victims and victimized exclusively in childhood or adulthood. Sexual revictimization was not associated with anxiety in C. Classen et al.’s (2002) study. Nevertheless, revictimized women had higher rates of simple phobia and social phobia than non-victims (Cloitre et al., 1997).

#### General psychological distress

Psychological distress consisted of different symptoms across studies, which might explain the inconsistent findings in 12 studies (*n* = 4 longitudinal).

The longitudinal studies reported that psychological distress mediated the relationship between CSA and prospective sexual victimization during adulthood (Gidycz et al., 1993; Orcutt et al., 2005), the relationship between child physical abuse and prospective intimate partner violence (IPV) (Lindhorst et al., 2009), and the relationship between CM and prospective dating violence (Cascardi, 2016). Similarly, cross-sectional studies supported the mediating role of psychological distress in the relationship between CSA and coercive sexual assault in adulthood (Fortier et al., 2009), and the relationship between CSA and IPV in the last 6 months (Engstrom et al., 2008). On the contrary, psychological distress was not an intervening factor between CSA and adult sexual victimization in another study (Gidycz et al., 1995).

Comparative cross-sectional studies provided inconsistent results. Psychological distress was higher in revictimized people than victimized exclusively in childhood (Aosved et al., 2011; Heidt et al., 2005; Kaltman et al., 2005; Proulx et al., 1995) or than exclusively abused in adulthood, and non-victims (Aosved et al., 2011; Heidt et al., 2005). However, no difference between sexually revictimized women and sexually abused exclusively in childhood was found by Gibson and Leitenberg (2001).

### Dissociation

Thirteen studies (*n* = 4 longitudinal) assessed the impact of different aspects of dissociation (*n* = 7 trait; *n* = 3 peri-traumatic; *n* = 3 posttraumatic; *n* = 1 somatoform dissociation).

The longitudinal studies provided inconsistent results. Zamir et al. (2018) followed 80 women for 32 years. Trait dissociation at the age of 19 mediated the relationship between childhood victimization and prospective IPV. Trait dissociation was related to physical, but not sexual revictimization in another longitudinal study with a 6-year follow-up (Noll et al., 2003). However, it was not a mediator between CM and prospective adult physical/sexual victimization in a longitudinal study with a 6-month follow-up (D. A. Young, 2017) or a mediating/moderating factor between childhood/adolescent and prospective adult sexual victimization in a longitudinal study with a 10-week follow-up (Sandberg et al., 1999). Peri-traumatic dissociation was associated with sexual, but not physical, revictimization in a longitudinal study (Noll et al., 2003).

The results of cross-sectional studies were not convergent. Field et al. (2001) reported higher trait dissociation in sexually revictimized women than sexually victimized in childhood and DePrince (2005) indicated a significant relationship. However, trait dissociation severity was not related to revictimization in another study (C. Classen et al., 2002). Pre-traumatic dissociation was unrelated to revictimization in two studies (Hetzel & McCanne, 2005; Irwin, 1999a, 1999b).

Cloitre et al. (1997) showed that more people in the sexually revictimized group reported scores close to or above the clinical cut-off than in the groups with sexual abuse exclusively in adulthood or non-victims. This difference was not found by Kaltman et al. (2005), but the revictimization group was very small (*n* = 10). Dissociation directly following exposure to a traumatic event was not related to sexual victimization in adulthood (Kessler & Bieschke, 1999). Finally, Dietrich (2007) found a relationship between sexual revictimization by a partner and dissociative disorders symptoms severity, but not between sexual/physical revictimization by a partner and somatoform dissociation.

### Alcohol/Substance Use

Ten studies (*n* = 4 longitudinal) investigated the effect of substance/alcohol use. The longitudinal studies indicated inconsistent results. Lindhorst et al. (2009), following participants for approximately 2 years, showed that neither general alcohol use nor general marijuana use were significant paths from childhood physical abuse to prospective sexual victimization in adulthood. Valenstein-Mah et al. (2015), following participants for 30 days, found that total drinks per week and drinking consequences, that is, impaired control, risky behavior, and blackout, did not predict prospective sexual revictimization facilitated by alcohol/drug and non-alcohol related sexual victimization among people with CSA, but blackout drinking predicted sexual revictimization facilitated by alcohol/drug in people with adolescent victimization. Gidycz et al. (1995) showed that alcohol abuse was not an intervening variable between CSA and prospective adulthood sexual victimization. Alcohol/substance abuse was not a mediator in the relationship between victimization in adolescence and adulthood in another study (Culatta et al., 2017). The pathway to IPV went from negative family environment to CM, then to risky behavior in adolescence including alcohol/substance use, which in turn was associated with adolescent sexual victimization, leading to IPV in adulthood (Fargo, 2008).

Similarly, the results of the cross-sectional studies were inconsistent. Alcohol and substance abuse did not mediate the association between CSA and IPV in the last 6 months (Engstrom et al., 2008). Moreover, substance and alcohol abuse disorder (as diagnosis) did not mediate the relationship between CSA and adulthood sexual victimization (Messman-Moore & Long, 2002). Nevertheless, the pathways from CSA to alcohol use and then to alcohol-facilitated sexual victimization in adulthood were significant, but not from alcohol use to forcible sexual assault in adulthood (Mokma et al., 2016). Gidycz et al. (1995) showed that alcohol use was not an intervening factor between CSA and retrospective adulthood sexual victimization. Lifetime prevalence of substance abuse disorder was not higher in sexually revictimized men than sexually victimized in childhood/adulthood and non-victims (Arata, 1999b).

#### Summary: Mental health

The results regarding the role of specific psychological symptoms and general psychological distress are mixed. As shown in Supplemental Table S1, methodological differences including different populations, definitions of CM/adulthood victimization, study designs (longitudinal vs. cross-sectional), and measures can potentially explain the inconsistencies. In general, there is evidence that psychological symptoms increase the risk of revictimization although it is not well studied yet how these factors make CM survivors more vulnerable for further victimization. A discussion about findings on each psychological symptom is provided below.

##### PTSD

Differences in sample sizes, populations, and measures explain inconsistent findings in both longitudinal and cross-sectional studies. Nevertheless, the findings show the importance of PTSD symptoms as a risk factor for revictimization, particularly for sexual revictimization in women. PTSD might compromise risk detection and reaction due to hyperarousal and numbing, respectively (Messman-Moore & Long, 2003). In addition, using alcohol or risky sex as emotion regulatory strategies to alleviate PTSD symptoms might interfere with risk detection.

##### Depression

Most studies found an association between depression and revictimization. The studies that did not support this relationship either had small sample sizes or assessed only recent adulthood victimization rather than victimization throughout adulthood. Even though the mechanism linking depression to revictimization is not as clear compared to PTSD, it has been suggested that the use of sex to cope with depressive symptoms might be the linking mechanism (Miron & Orcutt, 2014).

##### Anxiety

Although some studies showed a link between anxiety and sexual revictimization exclusively in women, it is not clear if anxiety is a consequence or risk factor for revictimization due to cross-sectional design of the studies. In addition, anxiety can be explained by PTSD symptoms and might not explain revictimization beyond PTSD symptoms. Small sample sizes and various used measures explain the inconsistent findings on anxiety.

##### Dissociation

The inconsistent results on dissociation may result from the examination of different aspects of dissociation. In addition, the summarized studies assessed trait dissociation by Dissociative Experience Scale (Bernstein & Putnam, 1986), which measures both pathological and non-pathological dissociation. Pathological dissociation is associated with CM, while non-pathological dissociation is not (Irwin, 1999a, 1999b), which might explain the inconsistent results since higher scores on dissociation might be due to higher non-pathological dissociation in some samples.

### Risk-taking in Sexual Relationships

#### Number of sexual partners

Nine studies (*n* = 3 longitudinal) were conducted on the number of sexual partners. In a longitudinal investigation (Testa et al., 2010), following the participants from adolescence to young adulthood, adolescent sexual victimization was related to risky sexual behavior in adolescence including the number of sexual partners, which in turn was related to risky sexual behavior in young adulthood, leading to prospective sexual adult victimization. However, a longitudinal study, with a 17-year follow-up, did not provide support in favor of higher number of sexual partners in sexually revictimized women than sexually abused exclusively in childhood, but prostitution was three times more likely in the former group (West et al., 2000). Gidycz et al. (1995) did not provide evidence for the intervening role of the number of sexual partners between CSA and prospective adult sexual victimization in a 9-month follow-up.

Higher number of sexual partners was consistently related to sexual revictimization in cross-sectional studies, except for one study (Gidycz et al., 1995). Sexually revictimized women reported a higher number of sexual partners (Arata, 2000) and higher sexual activity (Mayall & Gold, 1995) than women sexually abused exclusively during childhood. The number of sexual partners and/or relationships was related to sexual revictimization (Arata & Lindman, 2002) and it mediated the relationship between CSA and sexual victimization in adulthood (Arata, 2000; Santos-Iglesias & Sierra, 2012). In addition, Messman-Moore et al. (2010) indicated that the number of sexual partners was an intervening variable between CM and sexual victimization in adulthood. Ullman and Vasquez (2015) found a significant relationship between sexual revictimization and number of partners, but this variable did not mediate the relationship between childhood and adulthood victimization. Gidycz et al.’s (1995) study did not support the intervening role of the number of sexual partners between CSA and retrospective adult sexual victimization.

#### Sex under the influence of alcohol or substance

Of the six studies on sex under the influence of alcohol or other substances (SIAS), one was longitudinal (Krahé & Berger, 2017) and reported evidence for an association between CSA and SIAS (as well as other risky sexual behavior such as sex with a stranger), which in turn was related to prospective sexual abuse during adulthood.

Messman-Moore et al. (2010) measured the frequency of risky sexual behavior, that is, SIAS or sex without protection in a cross-sectional study. CSA was related to risky sexual behavior with regular partners, which in turn was associated with sexual victimization in adulthood. The same model included significant paths for sexual relations with strangers, with the difference that emotion dysregulation preceded risky sexual behavior in this case. In two cross-sectional studies, SIAS did not mediate the relationship between sexual abuse in childhood and adulthood (Santos-Iglesias & Sierra, 2012; Ullman & Vasquez, 2015), while in another study, using alcohol on dates was related to sexual revictimization (Arata & Lindman, 2002). Interestingly, Walsh et al. (2013) showed that CSA was negatively related to perceived control over sexual feelings, behaviors and interactions, which in turn was associated with the expectancy to enjoy sex more under the influence of substance/alcohol, which in turn was associated with alcohol/substance-facilitated sexual assault, but not forcible sexual assault.

#### Age at consensual sexual initiation

Three studies (*n* = 2 longitudinal) examined the initial age of consensual sex. One longitudinal study suggests that younger age at consensual sexual initiation mediates the relationship between CSA and physical, but not sexual abuse by partners during adulthood among women (Ihongbe & Masho, 2018). Among men, the mediating effects were neither significant for physical nor sexual abuse. Another longitudinal study (West et al., 2000) did not find any evidence for significant age differences at consensual sexual initiation in sexually revictimized students compared to peers sexually victimized in childhood and that result was also shown by a cross-sectional study (Kaltman et al., 2005).

#### Sex to reduce negative affect

Orcutt et al. (2005) discuss that the survivors of CSA might use sexual behavior for non-sexual goals such as a coping strategy to reduce negative affect or to feel powerful, which increase the risk of revictimization.

The three studies on this variable (*n* = 2 longitudinal) showed conflicting results. Miron and Orcutt (2014) supported a significant effect of sex to reduce negative affect on sexual revictimization in adulthood through depression and likelihood of sex with strangers over a time period of 57 days among university students. Conversely, Orcutt et al. (2005) did not find evidence for the mediating role of this variable in the relationship between sexual victimization in childhood and adulthood in a 5-year prospective study in a community sample. Having sex to receive love/attention or to deal with sadness/loneliness was associated with sexual revictimization in a cross-sectional study (Myers et al., 2006).

Relatedly, Reid (2009) found that sexual behavior/cognitions, such as using sex to control others and believing that men would not care about women without sex, in conjunction with sexual victimization in adolescence, mediated the relationship between maternal childhood neglect and sexual victimization in adulthood.

#### Sexual Assertiveness and Self-esteem

Of the six studies (n = 2 longitudinal), two studies were on sexual self-esteem, three on sexual asssertiveness, and one on sexual permissiveness.

Krahé and Berger (2017) found that CSA was related to lower sexual self-esteem, which in turn predicted higher prospective sexual victimization in adulthood in women, but not in men. In another longitudinal study (Livingston et al., 2007), sexual assertiveness was a significant intervening variable between CSA and prospective adult victimization. Relatedly, a longitudinal study (Noll et al., 2003) indicated a significant association between sexual permissiveness and physical revictimization, but not for sexual revictimization. In addition, sexual preoccupation was related to sexual, but not physical, revictimization.

Cross-sectional studies showed comparable results. Van Bruggen et al. (2006) showed that lower sexual self-esteem was an intervening variable between CM and adulthood sexual victimization through risky sex behavior. Moreover, sexual assertiveness mediated the relationship between sexual abuse in childhood and adulthood, with higher assertiveness being associated with lower revictimization (Santos-Iglesias & Sierra, 2012). Ullman and Vasquez (2015) also found a negative association between sexual assertiveness and sexual revictimization.

#### Summary: Risk-taking in sexual relationships

In sum, the findings on the association between risky sexual behavior and revictimization, except for sexual assertiveness, are mixed. In addition to methodological differences, that is, various measures and pathway models consisting of different risk factors, the inconsistent results might also be due to the heterogeneity in sexual activity among CSA survivors. People with CSA seem to respond to this traumatic event in two ways: avoidant coping that results in low sexual activity, and self-destructive coping that leads to elevated risky sexual activity (Gewirtz-Meydan, 2022; Merrill et al., 2003) and thus sexual revictimization. Null findings in previous studies could be due to the combination of these two groups. Consistent results regarding the effect of sexual assertiveness on revictimization is promising for preventive programs and suggests that reaction to risk can influence the occurrence of revictimization. Further research is needed on SIAS and the motives behind risky sexual behavior, such as emotion and self-esteem regulatory motives. In addition, previous studies did not consider the context of intoxicated sex well that could result in contradictory results. For instance, the level of alcohol/drug consumption, one versus two parties being intoxicated, and type of substances (low-risk vs. high-risk substances) are among the factors that might influence the risk of sexual victimization.

### Coping Strategies, Emotion Regulation, and Alexithymia

Eight cross-sectional studies queried coping strategies, three emotion regulation, and two alexithymia. Filipas and Ullman (2006) reported that a higher number of women in the revictimized group compared to exclusively sexually abused in childhood indicated using maladaptive coping strategies to deal with CSA, that is, drug/alcohol use for coping, withdrawal from people and sexual contacts as coping. Sexually revictimized women showed higher escape, negotiation, instrumental action, and self-blame than non-victims (Proulx et al., 1995), higher levels of cognitive and anxious coping, such as rumination and irritability, than exclusively sexually victimized in adulthood (Arata, 1999a), and greater use of disengagement coping, any attempt to avoid or disengage, than sexually victimized in adulthood (Gibson & Leitenberg, 2001). Nevertheless, revictimized women were not different than sexually victimizied in adulthood based on expressiveness, avoidance, self-destructive behaviors (Arata, 1999a), and engagement coping (Gibson & Leitenberg, 2001), and not different than sexually victimized in childhood when coping strategies were measured as a general variable (Mayall & Gold, 1995).

Of the two studies (Fortier et al., 2009; Irwin, 1999a, 1999b) testing the mediating role of coping in CM–revictimization association, only Fortier et al. (2009) showed that disengagement coping was an intervening variable between CSA and coercive (but not forceful) sexual victimization. Draucker (1997) showed that the ability to find a meaning in negative events did not mediate the relationship between CM and adult victimization.

Three studies consistently showed that emotion dysregulation was associated with revictimization (Messman-Moore et al., 2010; Ullman & Vasquez, 2015; Walsh & DiLillo, 2011), either as an intervening variable in a path model to sexual adulthood victimization (Messman-Moore et al., 2010) or as a difference in group averages (Walsh et al., 2011). Regarding the dimensions of emotion dysregulation, revictimized women had higher levels of non-acceptance of emotions, non-awareness of emotions, and lack of emotional clarity than other groups and greater lack of impulse control than women sexually victimized exclusively during adulthood. Ullman and Vasquez (2015) reported that emotion dysregulation in response to the most serious sexual assault in the past year was negatively associated with sexual assertiveness, which in turn was negatively associated with sexual revictimization.

Bell and Naugle (2008) found that alexithymia was associated with sexual revictimization after controlling for PTSD severity, CM, and behavioral avoidance to emotions. Cloitre et al. (1997) reported that alexithymia was more prevalent in revictimized women than sexually victimized exclusively in adulthood and non-victims.

#### Summary: Coping strategies

Most findings showed that various maladaptive coping styles and emotion dysregulation are risk factors for revictimization, although different definitions used for the maladaptive strategies can explain the null findings in two studies. Nevertheless, the results are in line with the developmental theory of emotion regulation that assumes the role of family functions, such as parenting styles and emotional climate of a family, in the formation of emotion regulation in childhood (Morris et al., 2007). Since CM occurs in a context of a disturbed family (Higgins & McCabe, 2003; Patwardhan et al., 2017), maladaptive emotion-regulation strategies, driven from a disturbed family and CM, develop and increase the chance of revictimization probably through risky sexual behavior.

### Social Factors

In this section, we will discuss evidence on three social factors—social support (*n* = 5), disclosure of sexual abuse (*n* = 2), and interpersonal relationships (*n* = 7), which were exclusively studied cross-sectionally, except for a study that had both cross-sectional and longitudinal design.

Schumm et al. (2006) showed that revictimized women reported lower social support compared to women exclusively victimized in childhood or adulthood. Lau and Kristensen (2010) and Mayall and Gold (1995) reported no difference between sexually revictimized women and women sexually victimized exclusively in childhood based on perceived social support following CSA and parental support in childhood, respectively. In two studies (Draucker, 1997; Engstrom et al., 2008), social support did not mediate the relationship between CM and IPV.

Two studies investigated whether deciding to disclose CM to friends or relatives—and their respective reactions in response to this discloser—are associated with revictimization. While Simmel et al. (2012) indicated that disclosure was not related to sexual revictimization, regardless of subsequent action after the disclosure. Wager (2013) reported a significant relationship between sexual revictimization and negative reaction to disclosure.

Previous studies indicated that the revictimized group had greater interpersonal problems such as submissiveness and intimacy than subjects sexually abused exclusively in childhood and non-victims (Cloitre et al., 1997), greater non-assertiveness, social avoidance, and over-nurturance than subjects sexually victimized in childhood (C. Classen, 2001), greater hostile and controlling behavior than subjects victimized exclusively in childhood (Lau & Kristensen, 2010), and higher interpersonal sensitivity and hostility than non-victims or subjects first victimized in adulthood (Messman-Moore et al., 2000). In addition, Dietrich (2007) showed that sexual revictimization was associated with interpersonal relationship problems in women, but not in men. In another study (Gidycz et al., 1995), difficulties with socialization and assertiveness were not an intervening factor in the association between CSA and retrospective/prospective adult sexual victimization. Self-silencing, avoiding self-expression, and effort for pleasing one’s partner were also related to sexual revictimization (Arata & Lindman, 2002).

#### Summary: Social factors

Although interpersonal problems were consistently related to revictimization, the findings did not support the role of social support in revictimization, except for a study in low-income women (Schumm et al., 2006). Although it is very early to reach any conclusion due to limited number of conducted studies and methodological differences, lack of evidence on the role of social support might show the weak effects of social support on revictimization. In line with this hypothesis, a literature review on the association between social support and psychological symptoms did not provide strong support for the buffering effect of this factor (Alloway & Bebbington, 1987). Another study showed that negative interactions in the context of support, such as perceived disapproval and pressure, had stronger association with depression than positive interactions in such a context (MaloneBeach & Zarit, 1995). Nevertheless, the effect of social support in low-income women might indicate that social support can be important in specific conditions, in which financial and societal resources are limited. Finally, as summarized studies measured general social support, testing specific dimensions of social support, emotional, informational, and instrumental, might help clarify the role of social support.

### Attachment and Interpersonal Cognitions

#### Attachment styles, parental caring/bonding, and family function

Two studies (*n* = 1 longitudinal) examined the role of parental bonding/caring. Jankowski et al. (2002) indicated that paternal and maternal care/warmth were not associated with sexual revictimization. Conversely, Reid (2009) indicated that a poor mother–child relationship linked to childhood neglect was associated with sexual behavior/cognitions, which in turn were associated with adulthood sexual victimization.

Three cross-sectional studies investigated the role of attachment. Hocking et al. (2016) provided support for the mediating role of anxious attachment in the relationship between CM committed by parental figures and revictimization, but two studies (Gay et al., 2013; Irwin, 1999a, 1999b) did not find any of the attachment styles to be significant factors. Since the two latter studies did not specify the perpetrators of CM, attachment styles might be an important risk factor for revictimization if the perpetrators are attachment figures. A cross-sectional study showed that family functions of intimacy and authority were not related with sexual revictimization (Arata & Lindman, 2002). Since the majority of the sample in this study were perpetrated by caregivers, it is likely that the small effect size found in the study could be the reason behind the null finding.

#### Early maladaptive schemas and cognitive distortions/attributions

Early maladaptive schemas, that is, long-lasting themes consisting of cognitions, emotions, and bodily sensations that are developed in childhood (Riso et al., 2006), were studied by two cross-sectional studies. The association between CM and IPV was mediated by mistrust, self-sacrifice, and emotional inhibition schemas (Crawford & Wright, 2007) and disconnection/rejection schemas had a mediating role in the the association between child emotional abuse and IPV (Gay et al., 2013).

Five cross-sectional studies examined cognitive distortions. Cognitive distortions about interpersonal relationship such as rejection by others or unrealistic interpersonal expectations were related to sexual revictimization in men, but not in women (Dietrich, 2007). Lau and Kristensen (2010) found greater cognitive distortions, that is, fearful, scared, mistrusting, and shyness, in sexually revictimized than victimized exclusively in childhood. Causal attributions specific to CSA incidents and general negative events were not different in sexually revictimized women than sexually victimized in childhood (Mayall & Gold, 1995). Feelings of stigma and powerlessness in reaction to recent adult sexual victimization were higher in sexually revictimized women than sexually victimized in adulthood, but not feeling of betrayal or beliefs about benevolence and meaninglessness of the world (Gibson & Leitenberg, 2001), and beliefs about externality or internality of reinforcement (Bolstad & Zinbarg, 1997).

##### Summary: Attachment and interpersonal cognitions

In general, the summarized studies found an association between negative schemas/cognitions, particularly for interpersonal cognitions and revictimization. Since there is overlap between attachment styles and early maladaptive schemas, that is, both are internal working models that guide how we see relationships and react to others, further insight into which specific attachment styles might be related to which specific schemas is important. Furthermore, future studies could examine if the protective effect of secure attachment style on revictimization depends on whether the perpetrator of CM includes a known or unknown person to a child.

### Self-blame

Five studies (*n* = 1 longitudinal) investigated self-blame. In a longitudinal study with a 7-month follow-up, self-blame did not mediate the relationship between adolescent sexual victimization and prospective adulthood sexual victimization (Katz et al., 2010). However, when sexual assertiveness was entered into the model as an intervening variable between self-blame and adulthood sexual victimization, the pathways were significant.

Four cross-sectional studies returned diverging findings. Filipas and Ullman (2006) reported higher self-blame about CSA at the time of abuse and at the current time in sexually revictimized women than sexually abused exclusively in childhood. Despite this, self-blame was not significantly associated with sexual revictimization. Arata (1999a) found higher characterological and societal self-blame regarding sexual assault incidents in sexually revictimized women than sexually victimized exclusively in adulthood, but no difference regarding situational self-blame. In addition, Arata’s (2000) results supported the mediating role of self-blame regarding CSA in the association between sexual victimization in childhood and adulthood. Mokma et al. (2016) showed that global self-blame was not associated with sexual revictimization among people with CSA. However, characterological and behavioral self-blame exhibited direct effects among CSA survivors. The pathways from global self-blame to alcohol use and to PTSD mediated the relationship between CSA and substance-facilitated revictimization.

#### Summary: Self-blame

The specific types of self-blame (global vs. specific) seem to be differently related to revictimization. Blaming one’s personal characteristics and behavior is related to revictimization. In addition, it seems that the linking mechanisms between self-blame and revictimization could be alcohol use, PTSD, and risky sexual behavior. However, one could argue that self-blame might be a cognitive distortion derived from different psychological symptoms, such as depression and PTSD, and it is not an additional risk factor beyond those symptoms. The different types of self-blame and the combination of different risk factors in interaction with self-blame in pathway models may explain the inconsistent results among the studies.

### Risk Detection

To our knowledge, this factor has not been studied longitudinally. Messman-Moore and Brown (2006) assessed risk detection in two risky dating scenarios. Women with sexual revictimization left the scenario with a stranger later than non-victims and showed higher discomfort in response to the sexual advances by a male acquaintance than women sexually abused exclusively in childhood. Moreover, DePrince (2005) tested the association between revictimization and detection of the violation of social contracts. The revictimized group had more errors on precaution (rules for keeping people safe) and social contract (rules for social exchange) problems than people with victimization exclusively in childhood. Pathological levels of dissociation were associated with these errors.

#### Summary: Risk detection

These findings, partially supporting poorer risk detection in revictimized people, should be interpreted cautiously as leaving a risky situation with delay might not necessarily reflect poor risk detection, but rather different (motivation-based) behavior. For instance, risky signals in a sexual encounter might be ignored as regulating negative emotions or boosting self-esteem with sex is given priority (Fereidooni et al., 2022; Miron & Orcutt, 2014; Myers et al., 2006). Therefore, interaction between motivations for risky behavior and risk detection needs to be investigated.

## Discussion

The purpose of the current systematic review was to integrate evidence on potential psychological mediators of revictimization. We identified 71 studies (*n* = 48 cross-sectional, *n* = 21 longitudinal, *n* = 2 mixed design) meeting our inclusion criteria. The summary of the findings and implications for future research are presented in Tables 1 and 2, respectively.

**Table 1.** Summary of the Findings.

1. Longitudinal and cross-sectional evidence indicates that psychological symptoms, particularly PTSD, are associates of revictimization.
2. Risk-taking sexual behavior is repeatedly related to sexual revictimization in cross-sectional studies, while the relationship in longitudinal studies is inconsistent.
3. Cross-sectional evidence shows that emotion dysregulation and pathological coping strategies are related to revictimization.
4. Prior cross-sectional research indicates that early maladaptive schemas and cognitive distortions are associated with revictimization.

**Table 2.** Implication for Future Studies.

1. Further longitudinal research is needed to understand if associates of revictimization are precursors or consequences of revictimization.
2. Interactions between associates of revictimization, such as relationship between emotion dysregulation and risky sex behavior, are not well studied yet and can be addressed in future research.
3. Further research on early maladaptive schemas, attachment styles, risk-taking sexual behavior, shame/blame, risk recognition, and social factors is needed.
4. Research on men, community samples and various cultures is limited and can be investigated in the future.

In the few available longitudinal studies, the following factors emerged as promising candidates for future studies: PTSD, general psychological distress, depression, and dissociation. Interventions focused on these psychological factors might decrease the risk of revictimization. Future studies should explore whether emotion regulation and coping strategies, such as using sex to reduce negative affect or alcohol/substance use, might be intervening factors between psychological symptoms and revictimization.

In conjunction, the reviewed cross-sectional studies presented convincing evidence that revictimized groups on average show increased levels of psychological symptoms as well as emotion regulation problems. However, due to the cross-sectional design of most studies, it remains unclear whether the observed differences are precursors or sequelae of revictimization. Emotion regulation difficulties, in turn, are associated with risk-taking in sexual relationships and alcohol consumption. Moreover, pathological forms of emotion regulation such as dissociation might affect proper risk detection or risk reaction such as assertiveness.

The available longitudinal studies on general substance/alcohol use, sex to reduce negative affect, and the number of sexual partners provided incongruent results. The differences in the samples, measures, durations of the studies, as well as level of sexual activity among CSA survivors might be the reasons for these inconsistencies. However, sexual revictimization was repeatedly associated with risk-taking sexual behavior in cross-sectional studies. Most of these studies investigated the impact of these factors separately, while different risky sex behaviors might interact with each other. For instance, sex to reduce negative affect in conjunction with intoxicated sex might have a stronger impact on revictimization.

Two studies showed that revictimized people are less aware of the violations of social rules by others and they have poorer risk detection. However, preliminary evidence on motives behind risky sexual behavior, using sex to reduce negative affect or to boost self-esteem, suggest that at least some revictimized individuals do not have more difficulties in recognizing risk signals, but instead they might intentionally disregard them in order to pursue other motivations in risky situations. Similarly, interactions between these risk factors might be also important in the context of interventions.

While several authors stipulate that altered attachment needs or schemas are central in mediating revictimization (Gold et al., 1999; Pilkington et al., 2021; J. E. Young, 2003), few studies to date showed that insecure attachment styles or early maladaptive schemas are related to revictimization. Furthermore, attachment needs might play even a more salient role in revictimization if CSA occurs within a family compared to CSA perpetrated by strangers. In addition, it remains unclear how insecure attachment or maladaptive schemas might influence behavior, which in turn might lead to revictimization. One could hypothesize that insecure attachment might be related to risky sex behaviors. For instance, anxious attachment might be associated with lower sexual assertiveness due to fear of rejection or avoidant attachment style might be related to higher number of sexual partners due to avoidance from intimacy. In addition, attachment styles and early maladaptive schemas might be related to revictimization via partner selection (Gobin, 2012).

It should be noted that of the 71 papers included in this research, 17 (23.9%) were published in the last decade (years 2012–2018), 38 (53.5%) in the decade before (years 2001–2011), and 16 (22.5%) between 19th century and 2000. We suggest this difference in publication rate found between 2012 to 2018 and 2001 to 2011 can be explained both by the difference in time periods covered (7 years vs. 11), as well as the fact that we found additional papers in the latter decade (2001–2011) by searching for eligible papers in the citations of found papers.

## Strengths and Limitations

The main strength of the current review lies in its systematic design, and including papers with similar definitions for revictimization based on developmental stage. Unlike previous reviews, we differentiated between different types of CM and adulthood victimization and did not include a specific form of victimization to reach to a general view about the risk factors of revictimization. The general perspective showed the risk factors for revictimization might depend on which population and types of victimization you test. It is important for future studies to include these factors.

As the scope of this study was not limited to sexual revictimization, we did not discuss the characteristics of CSA that might influence the risk of sexual revictimization. Factors such as frequency of victimization (single vs. multiple victimization), type of perpetrators (parents, strangers, intimate partners), and types of sexual contact (exhibitionism, fondling, intercourse) could be important to understand for whom these risk factors influence revictimization chances. Although the review included a variety of factors and a broad focus, some variables are overrepresented. For example, most studies focused on sexual revictimization, thus, other forms of revictimization, physical and emotional, should be considered in future research. In addition, most studies included heterosexual women and Caucasian student samples, particularly in the US, which makes it unclear if the findings are generalizable to other populations such as men, other non-heterosexual populations, and community samples.

Furthermore, different populations ranging from university students to inmates were studied, while risk factors for revictimization might vary among these populations. Thus, comparing all populations and not base conclusions on just a specific sample are crucial. Other limitations included cross-sectional designs and small sample sizes, various measures as well as different definitions for CM and adulthood victimization.

## Conclusion

The findings on most of the reviewed risk factors were inconsistent, which can be explained by methodological differences across the studies. Nevertheless, the results of this review allowed for drawing several conclusions. In summary, evidence shows that various psychological symptoms, risky sexual behavior, emotion dysregulation, and dissociation might be related to revictimization, but further research is still needed due to limited evidence. It is significant to examine how psychological risk factors interact with each other to predict revictimization. Studies on men, community samples and different cultures, and longitudinal research are among the gaps in the literature.

## Supplemental Material

sj-docx-1-tva-10.1177_15248380221150475 – Supplemental material for Childhood Maltreatment and Revictimization: A Systematic Literature Review Click here for additional data file. Supplemental material, sj-docx-1-tva-10.1177_15248380221150475 for Childhood Maltreatment and Revictimization: A Systematic Literature Review by Fatemeh Fereidooni, Judith K. Daniels and Miriam J. J. Lommen in Trauma, Violence, & Abuse
