# Supplementary material for: Childhood Maltreatment and Revictimization: A Systematic Literature Review
Source: Trauma Violence Abuse. 2023 Feb 3;25(1):291–305. doi: 10.1177/15248380221150475 (PMC10666465; doi:10.1177/15248380221150475)
Supplement: sj-docx-1-tva-10.1177_15248380221150475 – Supplemental material for Childhood Maltreatment and Revictimization: A Systematic Literature Review [file sj-docx-1-tva-10.1177_15248380221150475.docx]

**Table S1**

**Summaries of the included papers**

| **Author** | ***N*** | **Population** | **Mean _age_** | **Country (ethnicity)** | **Gender** | **Design** | **Definition of CM & adult victimization** | **Measures** |
| --- | --- | --- | --- | --- | --- | --- | --- | --- |
| Aosved et al., (2011) | 1,002 | U students | 20.31 | USA (mostly Caucasian) | M | C | CSA: sexual contact by a relative or someone 5 years older or by someone using threat of or actual physical force before 17. ASV: unwanted sexual contact by use of threat or force, use of alcohol/substance resulting in inability to consent & verbal coercion after 17. | Life Experiences Questionnaire, Expanded SES, Symptom Checklist-90-R. |
| Arata (1999b) | 92 | U students | 24 | USA (mostly Caucasian) | F | C | CSA: any sexual contact before 14 with someone at least 5 years older. ASV: sexual victimization since 14. | Custom: CSA, non-sexual interpersonal/non-human trauma, SES, SCID-Non patient edition. |
| Arata (1999a) | 119 | U students | 23 | USA (mostly Caucasian) | F | C | CSA: sexual contact with someone 5 years older before 14. ASV: forced penetration/ intercourse since 14. | Questions based on Finkelhor’s (1979) definition of CSA, SES, Sexual Assault Rating Scale, How I deal with Things, Trauma Symptom Checklist-33. |
| Arata (1999c) | 41 | U students | 24 | USA (mostly Caucasian) | F | C | CSA: sexual contact before 14 with someone at least 5 years older or with physical force/threat regardless of age difference. ASV: unwanted sexual contact subsequent to force, use of authority or intoxication since 14. | Questions based on Finkelhor's (1979) definition of CSA, SES, SCID, Crime Related-Post Traumatic Stress Disorder. |
| Arata (2000) | 221 | U students | 22.4 | USA (mostly Caucasian) | F | C | CSA: sexual contact with someone at least 5 years older or with physical force/thereat before 14. Adolescence/adult SV: SV after 14. | Questions based on Finkelhor’s (1979) definition of CSA, SES, Impact of Event Scale, custom: self-blame about sexual assault incident. |
| Arata & Lindman (2002) | 341 | U students | 20.9 | USA (mostly Caucasian) | F | C | CM: physical/sexual/emotional abuse, emotional/physical neglect before 14. ASV: coerced or forced sex contact due to a man’s use of position or authority, & intoxication induced by the man since 14. | CTQ, Family of Origin Scale, Self Scale, SES, custom: dating behavior. |
| Banyard et al. (2001) | 174 | Children attending hospital due to CSA & a reason other than CSA | 31.55 | N.R | F | C | CSA: genital contact documented in a hospital or retrospective report of sexual victimization before 13 or retrospective report of non-consenting/forced sexual contact between14 & 17. ASV: unwanted sexual assault ranging from fondling to penetration after 17. | Trauma Symptom Inventory, custom: CSA & ASV. |
| Bell & Naugle (2008) | 104 | U students | 20.42 | USA (mostly Caucasian) | F | C | CSA: sexual contact before 14 by someone at least five years. ASV: attempted or completed sexual assault after 14. | CTQ, Childhood Maltreatment Interview Schedule, SES, Images from Japanese & Caucasian Facial Expressions of Emotion & Neutral Faces, Modified PTSD Symptom Scale, Toronto Alexithymia Scale. |
| Bolstad & Zinbarg (1997) | 117 | U students | N.R | USA(mostly Caucasian) | F | C | CSA: sexual assault from exhibitionism to vaginal or anal intercourse by someone at least 5 years older by 14. ASV: unwanted sexual contact since 15. | Custom: CSA, ASV, Posttraumatic Stress Disorder Symptoms Scale-Self Report, I-E Scale. |
| Cascardi (2016) | 532 | Children from Child Protective Services | T1: approx. 14, T2: approx. 15.5, T3: 17-20 | USA (60% Hispanic, approx. 53% Caucasian) | F | L | CM: psychological & physical maltreatment, witnessing violence. AV: physical dating violence | CTS Parent–Child, Violence Exposure Scale for Children, CTS, Youth Self Report, Children’s Depression Inventory, Trauma Symptom Checklist for Children. |
| Classen et al., (2001) | 52 | Clinical sample with PTSD attending group therapy | Approx. 38 | USA (mostly Caucasian) | F | C | CSA: at least two incidents of sexual abuse involving genital contact between 3 & 15 with someone at least 5 years older, having been in a relationship with the perpetrator before the incident. ASV: sexual coercion, attempted rape, or rape in the last 6 months. | SES, Inventory of Interpersonal Problems. |
| Classen et al. (2002) | 58 | GP with a history of CSA | 40 | USA (mostly Caucasian) | F | C | CSA: at least two incidents involving genital contact between 3 & 15 with someone at least 5 years older. ASV: unwanted sexual contact, attempted & completed rape after 17. | Using interview for CSA, SES, Clinical Administered PTSD Scale for DSM-IV, Trauma Symptom Checklist-40, Stanford Acute Stress Reaction Questionnaire. |
| Cloitre et al., (1997) | 56 | GP | 33.5 | USA (mostly Caucasian) | F | C | CM: CSA i.e., at least one incident of sexual contact before 18 by a family member, caretaker or trusted adult 5 years older or incidents involving coercion regardless of age difference, physical abuse i.e., at least one incident of cruel & unusual punishment by parents or care givers. ASV: completed or attempted forced sexual contact since 18. | Child Maltreatment Interview Schedule, Sexual Assault History Initial Interview Schedule, Toronto Alexithymia Scale, Dissociative Experiences Scale, Inventory of Interpersonal Problems, SCID-III-R. |
| Crawford & Wright (2007) | 301 | U students | 20.37 | USA (Caucasian) | M (*n* = 143), F (*n* = 158) | C | CM: emotional abuse /neglect, physical abuse/neglect, & sexual abuse before 15. AV: physical, emotional, & sexual IPV. | Children of Alcoholics Screening Test, Lifetime Experience Questionnaire, Index of Dating Abuse, Young Schema Questionnaire. |
| Culatta et al., (2017) | 1,171 | U students | N.R | USA (mostly Caucasian) | F | L | Adolescence SV: attempted or completed sexual assault since 14. ASV: attempted or completed sexual assault since 14 during the study. | SES, Depression subscale of the Mental Health Inventory, custom: substance use. |
| DePrince (2005) | 116 | U students | 20.37 | USA (N.R) | M (*n* = 30), F (*n* = 86) | C | CM: Interpersonal violence before 18. AV: Interpersonal violence after 18. | Brief Betrayal Trauma Survey, Wason Selection Task, Dissociative Experience Scale, Trauma Symptom Checklist-40, Pathological dissociation (PD) taxon: based on Waller & Ross (1997). |
| Dietrich (2007) | 207 | Inmates, GP in counselling centers or via the internet | 37.9 | Canada (mostly Caucasian) | F (*n* =135), M (*n =* 87) | C | CM: psychological, physical, & sexual abuse, parental physical & psychological unavailability, parental substance abuse, & witnessing domestic violence by 17. AV: psychological abuse, physical & sexual abuse after 17. | Child Maltreatment Interview Schedule, Modified Child Maltreatment Interview Schedule, Detailed Assessment of Adult Posttraumatic Stress, Multiscale Dissociation Inventory, Cognitive Distortions Scale, Somatoform Dissociation Questionnaire. |
| Draucker (1997) | 622 | GP | 41 | USA (mostly Caucasian) | F | C | CM: psychological & physical maltreatment before 16, sexual contact with someone at least 5 years older. AV: emotional, physical & sexual abuse. | Psychological Maltreatment Scale, Physical Maltreatment Scale, Severity of Childhood Sexual Abuse Scale, Adult Victimization Scale–Custom, Revised Meaning Subscale of the Cognitive Adaptation Scale, Coopersmith Self-Esteem Inventory, Part 2 of the Personal Resource Questionnaire, Current Abuse Scale. |
| Engstrom et al., (2008) | 416 | Women in the treatment for methadone use | 39.9 | USA (mostly Latin/Hispanic & African-American ) | F | C | CSA: sexual experience from exhibitionism to any sexual contact prior to 15 by a relative or someone at least 5 years older or using force. AV: sexual, physical or psychological IPV in the last 6 month. | Childhood Sexual Abuse Interview, CTS-R, Posttraumatic Stress Diagnostic Scale, Brief Symptom Inventory, Drug Use and Risk Behavior Questionnaire, Multidimensional Scale of Perceived Social Support. |
| Fargo (2008) | 147 | People with documented history of CSA & without CSA attending a hospital | 31.6 | USA (mostly African-American) | F | L | CSA: any with someone at least 5 years older by 12. Adolescence SA: unwanted sexual contact from 13 to 17. ASV: unwanted sexual contact since 18. | Custom: CSA, adolescence SV, ASV, risk taking behavior/risky sexual behaviors, childhood family environment, Michigan Alcohol Screening Test. CTS. |
| Field et al. (2001) | 51 | GP sample with a history of CSA & diagnosis of PTSD | 38.4 | USA(mostly Caucasian) | F | C | CSA: at least two incidents involving genital contact between 3 & 15 with someone at least 5 years older, benign in a relationship with the perpetrator prior to the abuse. ASV: sexual coercion, attempted or completed rape in the last 6 months. | SES, Trauma Symptom Checklist 40, Modified Stroop Task. |
| Filipas & Ullman (2006) | 577 | U students | 19.6 | USA (Caucasian, Asian- American , Hispanic & African -American) | F | C | CSA: sexual contact before 14 with someone at least 5 years older. ASV: non-consensual intercourse after 14. | Questions based on Finkelhor's (1979) definition of CSA, Posttraumatic Stress Diagnostic Scale, custom: attribution of blame, SES. |
| Fortier et al. (2009) | 99 | U students | 21 | USA (mostly Caucasian) | F | C | CSA: sexual contact with a family member or someone at least 5-years older before 14, or with someone at least 10 years older or any unwanted sexual contact regardless of age difference or relationship with perpetrators between 14 & 17. ASV: sexual contact with coercion & aggression/force since 18. | Computer Assisted Maltreatment Inventory, Coping Strategies Inventory, Trauma Symptom Checklist-40, SES, Coping Strategies Inventory. |
| Gay etal., (2013) | 396 | U students | 19.14 | USA (mostly Caucasian) | F | C | CM: emotional abuse before 18. AV: psychological, physical & sexual IPV. | CTQ, CTS–R, Relationship Styles Questionnaire, Young Schema Questionnaire–Short Form. |
| Gibson & Leitenberg (2001) | 1050 | U students | 18.40 | USA (mostly Caucasian) | F | C | CSA: sexual contact before 14 with someone 5 years older or forced sexual activity, regardless of age difference. Adolescence SA: unwanted sexual contact from 14 to 17. ASV: unwanted sexual contact due to physical force, threat or intoxication in the last year. | Childhood Sexual Experiences Scale, SES , Stigma, Betrayal, Powerlessness & Self-Blame developed by Coffey, Leitenberg, Henning, Turner, & Bennett (1996), Brief Symptom Inventory, PTSD Symptom Scale-Self-Report, The World Assumptions Scale. |
| Gidycz et al., (1993) | 857 | U students | N.R | USA (mostly Caucasian) | F | C & L | CSA: sexual abuse ranging from exhibitionism to intercourse before 14, Adolescence SA: sexual abuse ranging from exhibitionism to intercourse since 14 & before university years. ASV: sexual assault during the study. | Custom: CSA, SES, Beck Depression/ Anxiety Scale. |
| Gidycz at al., (1995) | 796 | U students | N.R | USA (mostly Caucasian) | F | C & L | CSA: sexual abuse ranging from exhibitionism to intercourse before 14, Adolescence SA: sexual abuse ranging from exhibitionism to intercourse since 14 & before university years. ASV: sexual abuse during the study. | Custom: CSA, alcohol use, number of sexual partners, SES, Beck Depression/ Anxiety Scale, Inventory of Interpersonal Problems. |
| Heidt et al., (2005) | 342 | Homo & bisexual men & women | 32.06 | USA (mostly Caucasian) | M & F | C | CSA: Sexual contact before 18.  ASV: unwanted sexual experiences after 18. | Life Experiences Questionnaire Modiﬁed, SES-Modified, Brief Symptom Inventory, Beck Depression Inventory-II, Posttraumatic Stress Diagnostic Inventory. |
| Hetzel & McCanne (2005) | 467 | U students | 19.8 | USA (Mostly Caucasian) | F | C | CM: CSA i.e., sexual experiences ranging from an invitation to a sexual activity to completed intercourse before 15 with someone at least 5 years older, physical abuse before 15 with someone at least 5 years older. ASV: forced or unwanted sexual contact after 15, adult physical abuse after 15. | Childhood Sexual Experiences Questionnaire, Childhood Physical Experiences Questionnaire, Peri-traumatic Dissociation Experiences Questionnaire-Self-Report Version, Self-report version of the PTSD Interview. |
| Hocking et al., (2016) | 601 | U students | N.R | USA (mostly Caucasian) | F (71.4 %), M (28.6 %) | C | CM: negative home environment, punishment & sexual abuse before 14. AV: traumatic events after18. | Child Abuse and Trauma Scale, Experiences in Close Relationship, Betrayal Trauma Survey. |
| Ihongbe & Masho (2018) | 1,163 | U students | T1:  15.7; T3: 28.8 | USA (mostly Caucasian) | M (*n* = 335), F (*n* = 512) | L | CSA: sexual contact with parents or other adult caregivers by 6^th^ grade. AV: physical & sexual IPV. | Custom: CSA, IPV & initial age of sex. |
| Irwin (1999) | 155 | GP | 38.2 | Australia | F | C | CM: emotional abuse/neglect, physical abuse/neglect & sexual abuse before 14. AV: emotional & physical abuse since 18. | CTQ, Peri-traumatic Dissociative Questionnaire, Ways of Coping Questionnaire-R, Relationship Scales Questionnaire, SES, Physical Assault Scale, Injury Scale, CTS-R, Bad Things Scale. |
| Jankowski et al., (2002) | 974 | U students | 18.5 | USA (mostly Caucasian) | F | C | CM: genital contact before 16 with someone at least 5 years older or with physical force, physical injuries by parents before 16, witnessing interpersonal physical conflict among parents before 16. ASV: non-consenting sexual contact with threat or physical force after 16. | Childhood Sexual Experience Scale, Physical Aggression Scale of CTS, Parental Caring subscale of Parental Bonding Instrument. |
| Kaltman etal., (2005) | 125 | U students | 19.29 | USA (mostly Caucasian) | F | C | CSA: molestation or penetration before 12 by someone at least 5 years older. Adolescence SV: molestation or perpetration since 12 years, physical force or threat not required for relatives as perpetrators but required for non-relatives. | Russell’s (1986) Semi-structured Sexual Abuse Interview, Potential Stressful Events Interview, CTS, Stressful Life Events Screening Questionnaire, Trauma Symptom Inventory, Potential Stressful Events Interview, Structured Interview for Disorders of Extreme Stress, Structured Clinical Interview for DSM-IV–Non-patient version, Structured Clinical Interview for DSM-IV Personality Disorders, Symptom Checklist-90-R, Dissociative Experiences Scale, Sexual Functioning Scale, Social Adjustment Scale–Self Report. |
| Katz et al., (2010) | T1: 93, T2:87 | U students | 18.01 | USA (mostly Caucasian) | F | L | Adolescence SA: sexual contact since 15. ASV: sexual abuse during the study. | SES, Behavioral and Characterological Self-Blame Scale, Sexual Assertiveness Scale. |
| Kessler & Bieschke (1999) | 548 | U students | 21 | USA (mostly Caucasian) | F | C | CSA: sexual contact by 16. ASV: unwanted sexual contact or rape. | Childhood Experiences Questionnaire, Internalized Shame Scale, Trauma Symptom Inventory, SES. |
| Krahé & Berger (2017) | T1: 2,251,  T2: 1,612 | U students | 21.3 | German | F (*n* = 920), M (*n* = 1,331) | L | CSA: any sexual contact before 14. ASV: unwanted sexual contact without penetration, attempted or completed non-consensual penetration since 14. | Custom: CSA, risky sex behavior, Sexual Aggression and Victimization Scale, Sexual Self-Esteem Scale, Skill and Experience Scale. |
| Lau & Kristensen (2010) | 161 | Clinical sample | 33.3 | Denmark (N.R) | F | C | CSA: intra-familial CSA before 16. ASV: rape in adolescents or adulthood by a person other than the CSA offender. | Childhood Sexual Abuse Questionnaire, DSM-IV & ICD-10 Personality Questionnaire, Global Assessment of Functioning, Symptom Checklist-90-R, Cognitive Distortion from SCL-90-R. |
| Lindhorst et al., (2009) | 240 | Pregnant adolescents from public & private hospital prenatal clinics, public school alternative programs & social service agencies | T1: 16.6, T2: 20.9, T3: 22.4, T4: 22.9 | USA (mostly Caucasian & African-American) | F | L | CM: physical abuse by parental or guardians before 18. AV: physical IPV after 18, unwanted sexual contact due to psychological/physical force or threat after 18. | CTS- Modified, Symptom Checklist-90-R, custom: alcohol & marijuana use. |
| Livingston et al., (2007) | 937 | GP | 23.76 | USA (mostly Caucasian) | F | L | CSA: any sexual contact before 14. ASV: unwanted sexual contact since 14. | Items adapted from Finkelhor (1979) & Whitmire wt al., (1999) for CSA, SES, Sexual  Assertiveness Scale, Items adapted from National Women Study and Diagnostic Interview Schedule for depression, National Women’s Study PTSD Module. |
| Mayall & Gold (1995) | 654 | U students | 18.95 | USA (mostly Caucasian) | F | C | CSA: Sexual contact with someone 5 years older before 15. ASV: any unwanted sexual contact forced by verbal coercion, threat with weapon or physical force after 15. | Childhood Sexual Experience, custom: sexual activity, attributions about CSA, Parental Support Scale, How I deal with things Scale, Attributional style Questionnaire. |
| Messman-Moore et al., (2000) | 48 | U students | 19.74 | USA (mostly Caucasian) | F | C | CSA: sexual contact involving threat or force before 17. AV: unwanted intercourse with verbal or physical force, use of authority or alcohol/drugs intoxication, physical abuse since 17. | Life Experiences Questionnaire, Modified SES, CTS, SCL-90-R. |
| Messman-Moore & Long (2002) | 300 | GP | 37.4 | USA (mostly Caucasian) | F | C | CSA: sexual contact before 17 by a relative or someone more than 5 years older or due to threat/physical force. ASV: coerced intercourse due to pressure or misuse of authority, use of physical force or lack of resistance/consent due to intoxication since 17. | Life Experiences Interview, Structured Clinical Interview for DSM-IV-Non patient Version, Modified SES. |
| Messman-Moore et al., (2005) | 339 | Ustudents | 19.18 | USA (mostly Caucasian) | F | L | CSA: unwanted sexual contact before 17 by a relative or someone 5 years older or sexual contact with threat/force regardless of the age difference. ASV: rape after 17. | Life Experiences Questionnaire, SES, Trauma Symptom Inventory. |
| Messman-Moore & Brown (2006) | 262 | U students | 19.18 | USA (mostly Caucasian) | F | C | Child SA: sexual contact prior to 17 by a relative or someone 5 years older, or regardless of age difference if threat or force was involved. ASV: any unwanted sexual contact or  rape due to threats, use of force, inability to consent due to intoxication. | Life Experiences Questionnaire, Modified SES, Trauma Symptom Inventory, Risk Perception Survey. |
| Messman-Moore et al., (2009) | 276 | U students | 19.18 | USA | F | L | CSA: sexual contact by a relative or someone at least 5 years older or with threat or force before 17. ASV: rape i.e., oral, anal, or vaginal penetration through force/threat, or inability to consent due to intoxication after 17. | Life Experiences Questionnaire, CTQ, SES, Trauma Symptom Inventory, Drinking Habits Questionnaire, Alcohol Expectancy Questionnaire. |
| Messman-Moore et al., (2010) | 752 | U students | 18.76 | USA (mostly Caucasian) | F | C | CM: sexual contact with family members or someone at least 5 years older or unwanted sexual contact regardless of age difference before 14, physical violence by a parent or caregiver before 18. ASV: unwanted penetration due to threat/physical force or intoxication since 14. | Computer Assisted Maltreatment Inventory, SES, Difficulties in Emotion Regulation Scale, Cognitive Appraisal of Risky Events. |
| Miron & Orcutt (2014) | T1:1,043, T2:939, T3: 541 | U students | 19.6 | USA (mostly Caucasian) | F | L | CM: physical/emotional abuse by 14, sexual contact by 12. Adolescence SV: unwanted sexual experiences from 13 to 18. ASV: unwanted sexual experiences since 18. | Childhood History Questionnaire, Family Experiences Questionnaire, Traumatic Life Events Questionnaire, Depression Anxiety Stress Scale – 21, Motivations for Sexual Intercourse Scale, custom: likelihood of sex with strangers. |
| Mokma et al., (2016) | 929 | U students | 18.89 | USA (mostly Caucasian) | F | C | CSA: sexual contact before 17 with someone at least 5 years older. ASV: unwanted sexual experience via verbal coercion, intoxication or force after 17. | Life Experience Questionnaire, Modified SES, Cognitive Distortion Scale, Self-Blame Scale, Trauma Symptom Inventory, Drinking Habits Questionnaire. |
| Myers et al. (2006) | 147 | HIV-positive with a CSA | 39.33 | USA (mostly African-American & Hispanic) | F | C | CSA: any sexual contact with someone at least 5 years older before 18. ASV: attempted or completed rape after 18. | Custom: CSA, ASV, risky sex behavior, PTSD Diagnostic Module of University of Michigan version of Composite International Diagnostic Interview, Trauma Symptom Inventory, Center for Epidemiological Studies–Depression Scale. |
| Noll et al., (2003) | 140 | Children with CSA referred by protective service agencies & a individuals from GP | T1: 11.11 | USA ( approx.. 50% Caucasian, 50% minorities) | F | L | CSA: documented sexual contact by a family member before 14. AV: sexual  contact and/or rape or attempted rape by a nonfamily member since 14, beaten up, mugged, physically hurt  by a partner or being seriously hurt in any other way after 14. | Comprehensive Trauma Interview, Sexual Activities and Attitudes Questionnaire, Adolescent Dissociative Experiences Scale, Peri-traumatic Dissociative Experiences  Questionnaire Scale, Structured Interview for PTSD, |
| Orcutt et al., (2005) | T1: 1,033 T2 & T3: 800 | GP | T 2: 21.23 , T 3: 27.19 | USA (Mostly Caucasian & African-American) | F | L | CSA: unwanted sexual experiences with a family member before 16 or any sexual experience with a family member at least 5 years older, unwanted sexual experience with a nonfamily member before 14 or unwanted intercourse with a nonfamily member between 14 & 16. Adult SA: unwanted completed penetration by force or the threat of force btw T 2 & T 3. | Psychological Maltreatment Scale, Items from Wilsnack, Vogeltanz, Klassen, &Harris (1997) study for CSA, Family Experiences Questionnaire, Motives for Sexual Intercourse Scale, Brief Symptom Inventory. |
| Proulx et al., (1995) | 883 | U students | 18 | Canada (mostly Caucasian) | F | C | CSA: unwanted sexual contact by 16. ASV: unwanted sexual contact after 16. | History of Unwanted Sexual Contact, Ways of Coping Scale-R, Beck Depression Inventory, Taylor Manifest Anxiety Scale, Symptom Checklist-90-R. |
| Reid & Sullivan (2009) | 174 | Women with a history of official & self-report CSA | T1: 8.4, T3: 31.6 | USA (mostly African- American) | F | L | CM: child neglect e.g., poor supervision, inadequate food or medical care, & sexual contact with someone at least 5 years older before 13. Adolescence SV: unwanted genital contact from 13 to 17. ASV: unwanted sexual experiences since 18. | Official report & self-report CSA while attending to a hospital in childhood due to CSA or another reason, custom: child neglect, mother-child attachment, ASV, adolescence SV, shaming sexual beliefs & behaviors measured based on Siegel & Williams (2003) |
| Risser et al., (2006) | 1,449 | U students | 19 | USA (mostly Caucasian) | F | C | CSA: sexual experiences ranging from an invitation for sexual contact to intercourse before 15 someone at least 5 years older. ASV: forced or & unwanted sexual experiences after 15. | Finkelhor’s Survey of Childhood Sexual Experiences-R, Adult Sexual Experiences Questionnaire, PTSD Interview-Self-report. |
| Sandberg et al., (1999) | 323 | U students | N.R | USA(mostly Caucasian) | F | L | CSA/adolescence SA: sexual contact before 16 with someone at least 5 years older or with threat/physical force. ASV: any unwanted sexual contact after 15 & during the study | Childhood Sexual Victimization Questionnaire, SES, Impact of Event Scale-R, Dissociative Experience Scale. |
| Santos-Iglesias & Sierra (2012) | 402 | U students | 20.82 | Spain | F | C | CSA: sexual assault before 13.  ASV: unwanted, forced or intoxicated sexual intercourse after 14. | Juvenile Victimization Questionnaire, SES, Refusal Subscale of Spanish Validation of Morokoff’s Sexual Assertiveness Scale, custom: initial age of sex, number of sexual partners & sex under the influence of alcohol/substance. |
| Schumm et al., (2006) | 777 | Women in obstetric-gynecological clinics for low-income women | 21.7 | USA (mostly African-American) | F | C | CM: physical & sexual abuse before 16. ASV: physical & sexual abuse (e.g., penetration) since 16. | CTQ, Social Provisions Scale, Center for Epidemiologic Studies–Depression Scale, PTSD Symptom Scale–Self-Report. |
| Simmel et al., (2012) | 234 | Women in sexual assault & domestic violence agencies, GP not receiving services from the agencies, & women from the state prison | 36 | USA (approx. half Caucasian) | F | C | CSA: sexual experiences before 17. ASV: rape, attempted rape & sexual coercion. | Childhood Maltreatment Interview Schedule, SES, custom: disclosure/action following disclosure. |
| Testa et al., (2010) | 469 | High school students | T1: 18.1 | USA (mostly Caucasian) | F | L | Adolescence SA: unwanted sexual contact since 14. ASV: non-consenting sexual contact in the first year of college. | SES, custom: alcohol use & number of sexual partners or casual sex, items adapted from Read, Wood, & Capone (2005) for drinking contexts. |
| Ullman et al., (2009) | 555 | GP with a history of ASV | 31.01 | USA (approx. Half African-American, 38% Caucasian) | F | L | CSA: unwanted intercourse subsequent to verbal force or use of authority or unwanted fondling/kissing due to verbal/physical force or use of authority before 14. ASV: unwanted intercourse subsequent to verbal force or use of authority or unwanted fondling/kissing due to verbal/physical force or use of authority since 14. | SES, Posttraumatic Stress Diagnostic Scale, Michigan Alcoholism Screening Test. |
| Ullman & Vasquez (2015) | 1,094 | GP | 35 | USA (Mostly Caucasian & African-American) | F | C | CSA: sexual contact before 14. ASV: sexual assault since 14. | SES-R, Difﬁculties in Emotion Regulation Scale, Sexual Assertiveness Scale, risky sex behaviors items adapted from Campbell & colleagues (2004) study. |
| Valenstein-Mah et al., (2015) | 162 | U students | 20.21 | USA (mostly Caucasian) | F | L | CSA: sexual contact before 14 with someone at least 5 years older. ASV: completed or attempted penetration & unwanted sex due to intoxication after 14. | Childhood Sexual Victimization Questionnaire, Modiﬁed SES, Daily Drinking Questionnaire, Young Adult Alcohol Consequences Questionnaire. |
| Van Bruggen et al., (2006) | 402 | U students | 19 | Canada (mostly Caucasian) | F | C | CM: physical, emotional & sexual abuse before 14. ASV: unwanted sexual contact since 14. | Childhood Maltreatment Interview  Schedule-Short Form, SES, Trauma Symptom Inventory,  Socio-sexual Orientation Inventory, Sexual Self-Esteem Inventory for Women. |
| Wager (2013) | 481 | GP | 31.2 | UK (mostly Caucasian) | F (84.4%) & M | C | CSA: sexual contact ranging from watching sexual acts to penetration before 14. ASV: sexual contact ranging from watching sexual acts to penetration since 14. | Custom: CSA, ASV, reaction to disclosure. |
| Walsh et al., (2011) | 160 | Incarcerated women in a correctional facility | 35.4 | USA (mostly Caucasian) | F | C | CM: emotional abuse/neglect, physical abuse/neglect & sexual abuse before 14. ASV: forced or intoxicated sexual experiences since 14. | CTQ, SES-R, Difficulties in Emotion Regulation Scale. |
| Walsh at al., (2013) | 714 | U students | 19.7 | USA (mostly Caucasian) | F | C | CSA or adolescence SV: sexual contact before 18. ASV: unwanted sex due to intoxication or threat/physical force since 18. | CTQ, Computer Assisted Maltreatment Inventory, Modified SES, PTSD Checklist-Civilian. |
| Walsh et al. (2013) | 546 | U students | 18.7 | USA (mostly Caucasian) | F | C | CSA: sexual contact before 14.  ASV: unwanted sexual experiences due to threat/force or intoxication since 14. | CTQ, Modified SES, Risky Sex Scale, Sexual Self-Esteem Inventory for Women. |
| West et al., (2000) | 113 | People with documented history of CSA | T1: 10 months to 12 years, T2: 25.2 | USA (African-American) | F | L | CSA: sexual contact by force, misuse of authority with someone at least 5 years older.  ASV: unwanted sexual contact after 18. | Documented history of CSA resulting in attendance in emergency room,  Custom: ASV, risky sex behavior. |
| Young et al., (2017) | 300 | Homeless & unstable housed women | 46.99 | USA (N.R) | F | L | CM: physical violence & forced sex before 18. AV: physical & sexual abuse after 18: occurred within 6 months before baseline & at the follow-up. | Severity of Violence Against Women Scales, Dissociative Experiences Scale. |
| Zamir et al., (2018) | 80 | Children born to first-time mothers recruited at free public health clinics with mothers living below the poverty line | N.R | USA (mostly Caucasian) | F | L | CM: physical abuse i.e., physical force resulting in injury, & sexual contact or non-contact exploitation before 17.5. IPV: physical abuse between 20 & 32. | CTS, Dissociative Experiences Scale. |

ASV: adult sexual victimization, C=cross-sectional, CM: childhood maltreatment, CPA = Childhood physical abuse, CSA: childhood sexual abuse, CTQ = Childhood Trauma Questionnaire, CTS = Conflict Tactics Scale, Custom: Custom-made, GP = general population, F= Female, IPV: Intimate partner violence, M=Male, N.R = Not reported, R = revised, SES: Sexual Experience Survey, SV: sexual victimization, T = time, U students = University students
